# Are we training residents to communicate with low health literacy patients?

**DOI:** 10.3402/jchimp.v2i4.19238

**Published:** 2013-01-07

**Authors:** Nadia K. Ali

**Affiliations:** Department of Internal Medicine, Crozer Chester Medical Center, Upland, PA, USA

**Keywords:** health literacy, patient-provider communication, interpersonal and communication competency

## Abstract

**Introduction:**

A third of Americans have low health literacy (HL). Research indicates a significant knowledge and skills gap among residents pertaining to management of patients with low HL.

**Objectives:**

The objective of this study was to assess the teaching and evaluation methods around HL in community-based internal medicine residency programs. In addition, the study compared the teaching and evaluation practices used for doctor–patient communication skills to those used for HL skills.

**Method:**

A structured questionnaire was completed by faculty of community-based internal medicine residency programs through the ‘Community Hospital Education and Research Network’ website and the Association of Program Directors in Internal Medicine community hospital assembly meeting in October 2011.

**Results:**

Less than 50% of the programs provided any formal teaching on HL. HL was primarily taught via didactics (75%) followed by clinical observation (42%) and role-playing (25%). On the contrary, patient–provider communication skills were taught primarily using clinical observations (90%) and standardized patients (46.7%). The HL dimensions that programs focused on were the use of teach-back technique (100%), prevalence of low HL (83.3%), association between low HL and patient outcomes (83.3%), and use of plain language (83.3%). The areas that were least taught included helping patients navigate the health system (33.3%) and choosing effective written material for low HL patients (50%).

**Conclusion:**

Health literacy is not being taught consistently as part of the core curricula of the community-based internal medicine residency programs. There is a need for professional and technical resources to incorporate HL teaching in their curricula. There is a wide variation in terms of how HL skills are being taught and evaluated by community-based internal medicine residency programs.

On July 1, 2002, competency in interpersonal and communication skills was identified as one of the six required qualities to be taught, evaluated, and reported by each residency program to the Accreditation Council for Graduate Medical Education (ACGME) (1). There are over 90 million Americans with low health literacy (HL); therefore, an important aspect of the doctor–patient communication (DPC) is the ability to communicate with low HL patients who lack the skills to find, understand, evaluate, communicate, and use health-related information (2, 3). Low HL puts these individuals at risk of serious adverse outcomes such as increased frequency of emergency visits and hospitalizations and poor utilization of screening services (4). Elderly patients with low HL have higher mortality and report poor health status (4). Studies have shown that resident physicians are often unable to recognize patients with low HL (5). In addition, they are often unaware of the adverse outcomes associated with low HL and lack skills in counseling these patients (6). The issue of HL is a national priority, and best practices and resources are available to help providers communicate with low HL patients (7–10). Unfortunately, little is known about how residency programs are incorporating HL teaching in their core curriculum. This study was conducted to determine how HL was being taught and evaluated in the community-based internal medicine residency programs and to identify the resources needed by these programs to incorporate HL teaching in their core residency curriculum.

## Methods

### Sample

Program directors and associate program directors of over 90 accredited community-based internal medicine residency programs have collaborated to be part of a web-based network called ‘Community Hospital Education and Research Network’ (CHERN) (11). Around 40 of these institutions are part of the CHERN survey group. A cross-sectional web-based, self-administered survey was circulated through the CHERN website in December 2011. In addition, it was also distributed during the ‘Association of Program Directors in Internal Medicine’ (12) (APDIM – the international organization of accredited internal medicine residency programs) community hospital group assembly in October 2011.

### Survey

**Fig. 1:**
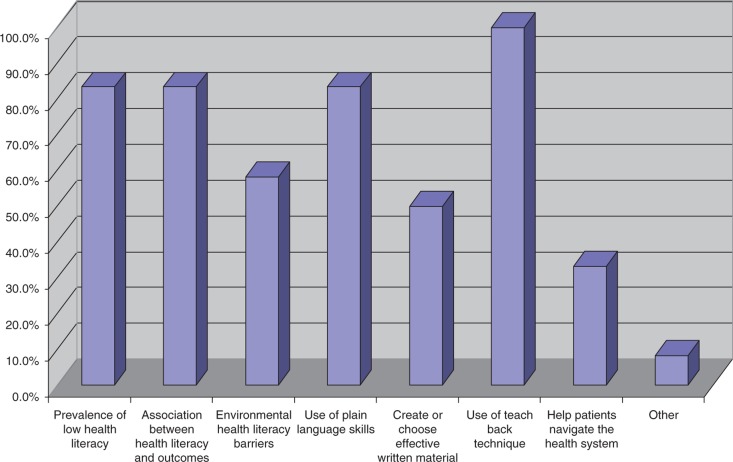
What areas pertaining to health literacy are taught in the core curriculum? (Check all that apply)

**Fig. 2:**
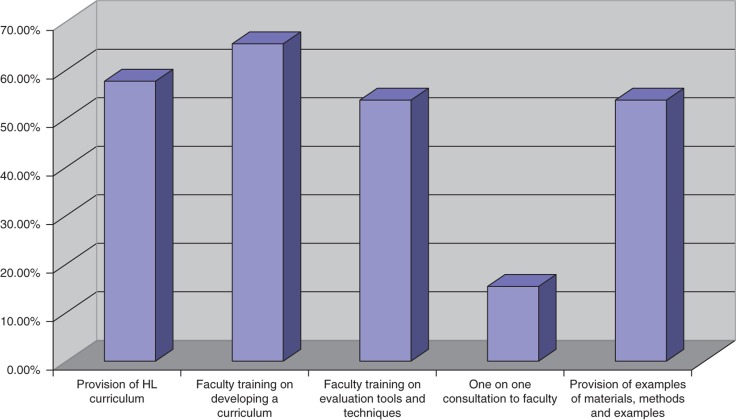
What type of support your program needs to introduce health literacy teaching in the core curriculum? (Check all that apply)

**Fig. 3:**
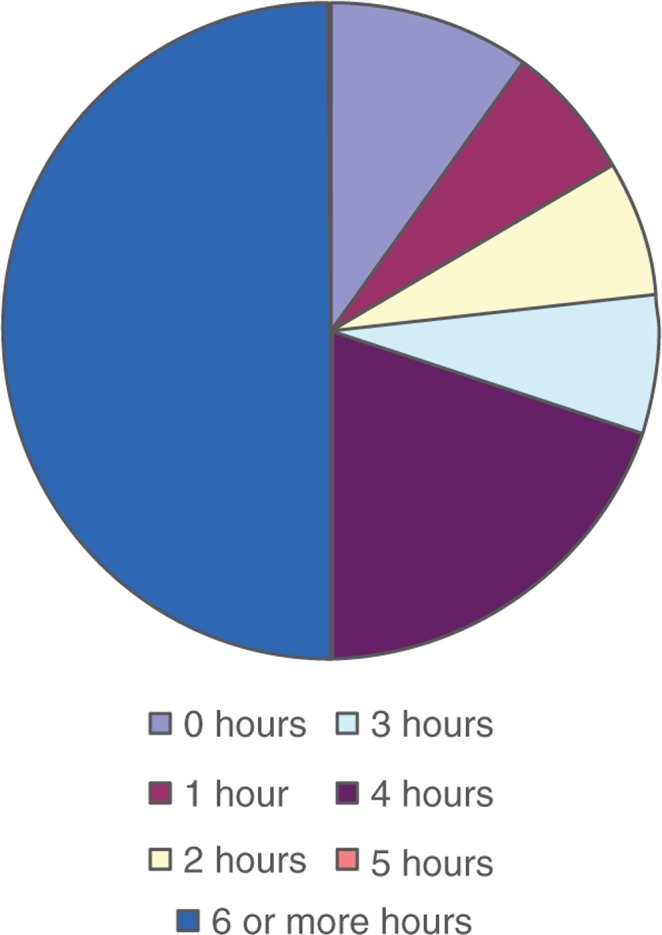
In the core residency curriculum at your institution, approximately how many hours of formal instruction are devoted specifically to health communication or doctor–patient communication?

**Fig. 4:**
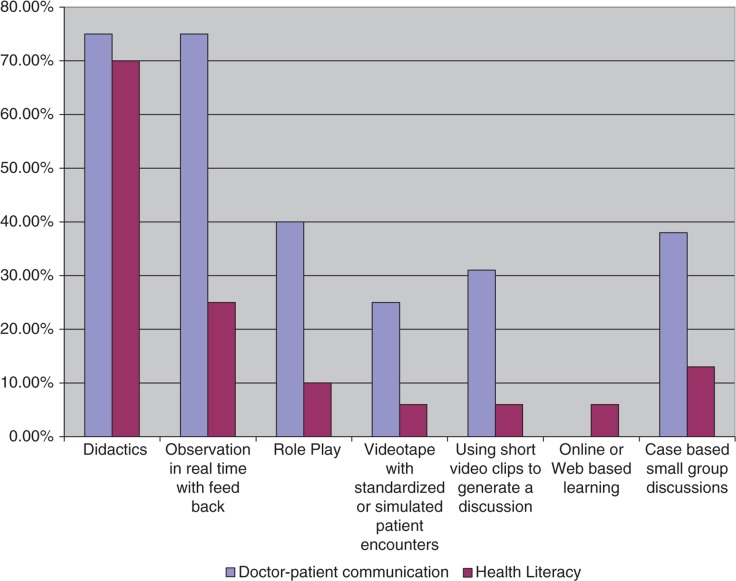
Comparison of teaching methods for doctor–patient communication and health literacy.

A 19-item validated survey tool developed by Oregon Health and Science University (OHSU) to assess the quantity and characteristics of HL teaching in US medical schools was tailored for obtaining information about practices around teaching HL from an internal medicine residency program faculty (13). The survey provided a brief explanation of the purpose of the study to the participants. It consisted of 10 items that included time assigned for formal teaching of DPC, incorporation of HL in the core curriculum, different methods used to teach and evaluate HL and DPC, the specific aspects of HL that are being taught, identification of the resources needed to teach HL, willingness of programs to share their experiences around HL teaching, and participation in the creation and design of a curriculum around HL. Figures 1–4 provide specific examples of the questions from the survey.

### Data analysis

Data analysis was carried out via SPSS version 20.0. Primary analysis was carried out by computation of descriptive data for each of the variables. Chi-square analysis was carried out for secondary analysis to calculate the association between the different items.

## Results

### Health literacy training

Thirty program directors and associate program directors completed the survey. Eleven surveys were completed online. Nineteen surveys were completed at the APDIM fall meeting.

Forty-three percent (*n*=13) of respondents reported teaching HL as part of the residency curriculum. The most common method of teaching HL was through didactics or lectures (*n*=9) followed by observations in real time with feedback (*n*=5) and role play (*n*=3). The HL teaching modalities that were least likely to be used included online or web-based learning (*n*=1), use of standardized patients (*n*=1), and short video clips to generate discussions around HL (*n*=2). Other teaching methods used to teach HL included provision of required reading to house staff, inviting guest speakers, and encouraging house staff to do a research project on HL.

The majority of the respondents focused their HL teaching on four content areas: using the teach-back or show me technique to check patients’ understanding (*n*=12), prevalence of low HL (*n*=10), association between HL and patient outcomes (*n*=10), and use of plain language skills (i.e., the ability to communicate with patients in lay terms, without the use of medical jargon) for verbal communication (*n*=10). The HL content areas that were least taught included helping patients navigate the health system (*n*=4), creating or choosing effective written (or printed) material for patients with low HL (*n*=6), and environmental HL barriers in the clinical setting (*n*=7) (see Fig. 1).

Respondents were using a wide variety of methods to evaluate the residents’ understanding of HL, including clinical observation, Mini-CEX, patient satisfaction questionnaires, standardized patients, multiple choice questions, and 360° feedback. The most common method used was clinical observation (*n*=8).

Assessment of the needs of the community-based programs around incorporating HL teaching in the core curriculum included training for their program faculty pertaining to development of a HL curriculum (*n*=17), curriculum on HL (*n*=15), faculty training around evaluation techniques and tools (*n*=14), and examples of materials, methods, and ideas used by other programs (*n*=14) (see Fig. 2).

### DPC training

The time spent providing formal teaching around DPC ranged from 0–3 hours to 6 or more hours (see Fig. 3). The teaching methods commonly used to teach DPC include observation in real time with feedback (*n*=22), didactics or lectures (*n*=19), and role play (*n*=16). The least used teaching method was online or web-based learning. The most common method for assessing residents’ understanding of DPC was clinical observation (*n*=27) followed by the use of standardized patients (*n*=14). Other less commonly used methods to assess understanding included multiple choice questions, writing a reflective piece, patient satisfaction survey, and 360° feedback.

### Comparison between HL and DPC training

Eleven of the 12 respondents that were using didactics as a teaching method for DPC were also using it to teach HL. However, only one third of the respondents that used observation in real time with feedback and role-play for teaching DPC were using that method for teaching HL. The teaching modality that is least used in DPC training and HL is online or web-based learning. See Fig. 4 for a comparison of the teaching methods used for DPC relative to HL. Chi-square tests did not reveal a significant difference between the use of the different teaching methods for DPC relative to HL: didactics χ^2^(1, *N*=16)=3.41, *p*=0.14; clinical observation χ^2^(1, *N*=16)=1.78, *p*=0.52; role playing χ^2^(1, *N*=16)=2.93, *p*=0.18; use of standardized patient encounters χ^2^(1, *N*=16)=0.76, *p*=0.45; use of short video clips χ^2^(1, *N*=16)=0.37, *p*=1.00; and case-based group discussions χ^2^(1, *N*=16)=1.34, *p*=0.52.

Eight of 10 respondents using clinical observation for assessing DPC were using the same method for assessing the understanding of HL. However, none of the respondents using standardized patients for evaluation of DPC were using this tool for assessment of HL skills. Multiple choice questions and writing a reflective piece were the least used evaluation methods for both DPC and HL. There was no significant difference between the use of different methods of evaluation between DPC and HL: multiple choice questions χ^2^(1, *N*=11)=0.11, *p*=1.00; clinical observation χ^2^(1, *N*=11)=0.24, *p*=1.00; and use of standardized patients χ^2^(1, *N*=11)=1.40, *p*=0.49.

## Discussion

This is the first published study that attempts to identify how community-based internal medicine programs are addressing HL in their core curriculum. An important limitation of this study is the small sample size, which may account for its inability to demonstrate differences in the methods of teaching and evaluating HL relative to DPC. It is not possible to draw absolute conclusions on the basis of this study. However, it is possible to review the trends and practices around HL teaching. Another limitation of this study was the cross-sectional nature of the study, which lends itself to both recall and reporting bias. The recall bias may occur if faculty does not recall all of the different teaching and evaluation methods that their program uses. In particular, this may be the case if the faculty member is not involved or interested in the subject under consideration. The reporting bias stems from a tendency of respondents to report positive or desirable results.

It is interesting to note that there is a lot of variation in terms of the methods being used to teach and evaluate HL as well as the resources identified by the faculty to incorporate HL in their core curriculum. Potential reasons for this variation could be the variation in the respondents’ residency program size as well as familiarity and experience around teaching DPC and HL.

Around 80% of the respondents reported that they spent 3 or more hours on the provision of DPC but less than 50% of respondents reported teaching HL as part of their core curriculum. This trend is definitely concerning because low HL is an epidemic faced by one third of all Americans (3). An important factor accounting for this gap can be explained by the lack of support expressed by the respondents in terms of both human and material resources. There are considerable resources and tools that have been developed and made available for teaching DPC skills (14, 15). These resources do not specifically address communication with low HL patients. The ACGME has recently developed milestones for each of the competencies, including interpersonal and communication skills, but there are no milestones that specifically address communication with low HL patients (16). Programs that teach HL are appropriately focusing on communication skills found to be effective for low HL patients such as teach-back technique and using plain language (17). This study did not focus on the time frame of the content delivery and also whether the HL teaching was delivered as a one-time teaching module or as a longitudinal curriculum. In addition, the study did not inquire as to whether the HL teaching was focused on interns or residents.

The teaching tool box to integrate communication skills in the medical curricula developed by the Harvard Macy Institute Program strongly recommended the use of experiential methods of teaching such as observation in real time with feedback and role modeling compared to didactic methods for teaching communication skills (15). Respondents in our study used didactic formats as well as experiential strategies such as standardized patients and role-playing for teaching DPC and HL. The study lacked the power to determine if there was a statistical difference between the uses of didactic versus experiential teaching strategies for DPC versus HL. However, there was a trend toward more experiential teaching strategies for DPC compared to HL.

The Miller's framework grades clinical competency from the lowest level of knowledge acquisition, followed by competence (know how to perform the skill), performance (demonstrate the skill), and finally the highest grade of action (performing in the actual setting) (18). More than 80% of respondents were appropriately using actions such as clinical observation with feedback or performance such as standardized patients to evaluate DPC and HL skills.

Although there have been various attempts to develop curricula around HL at various levels, such as medical schools, residency programs, and continuous medical education programs for physicians, there is no outcome data to indicate the best approach for teaching or evaluating HL skills (19). In addition, there is no consensus around what HL areas need to be included in a residency's core curriculum. This knowledge gap combined with lack of faculty development opportunities for learning the use of available teaching and evaluation tools around HL make it a challenge for community programs to introduce ‘health literacy’ teaching in their core curriculum.

## Conclusion

This study suggests that HL is not consistently being addressed in the core residency curricula of community-based internal medicine residency programs. Faculties of community-based internal medicine residency programs have indicated the need for human and material support in order to introduce HL teaching in their curricula. There is a need to develop HL-specific measurable objectives or milestones as part of the interpersonal and communication skills competency to encourage residency programs to incorporate HL in their core curriculum. In addition, there is a need for outcomes research on the existing HL curricula, teaching tools, and evaluation strategies. Finally, faculty development resources need to be made available to residency programs for effectively teaching and evaluating communication and HL skills.
